# Editorial: Cell adhesion molecules in neural development and disease

**DOI:** 10.3389/fnins.2022.1112300

**Published:** 2023-01-10

**Authors:** Robert Hindges, Zsolt Lele

**Affiliations:** ^1^Centre for Developmental Neurobiology, King's College London, London, United Kingdom; ^2^MRC Centre for Neurodevelopmental Disorders, King's College London, London, United Kingdom; ^3^Laboratory of Molecular Neurobiology, Institute of Experimental Medicine, Budapest, Hungary

**Keywords:** cell adhesion molecules, protocadherin, teneurin, N-cadherin, circuit assembly

Cell-to cell adhesion is a defining, hence essential condition of being a multicellular organism. It has been more than 60 years that Weiss published the first in a series of pioneering papers detailing various aspects of cellular adhesion (Weiss, 1959). Since then, a large number of papers has been published on this fascinating Research Topic describing all the studies that contributed to the state-of-the-art knowledge of today. In this Research Topic of Frontiers in Neuroscience, we collected a series of papers, both original research articles and reviews to emphasize the importance of cell adhesion molecules in neural development and disease. Two of the original research papers presents novel data involving protocadherins. Members belonging to this family have previously been demonstrated to be responsible for dendritic self-avoidance (Kostadinov and Sanes, 2015; Lefebvre et al., 2015; Ing-Esteves, 2018), axon sorting of olfactory sensory (Mountoufaris, 2017), and serotonergic neurons (Chen, 2017; Katori, 2017). In this Research Topic, Pancho et al. demonstrates the importance of PCDH19 in interneuron migration while Luo et al. propose the involvement of PCDH11x in target specification of hippocampal mossy fibers. An excellent overview provided by Moreland and Poulain outlining the role various cell adhesion molecules play in neural circuit assembly. As a perfect continuation of this Research Topic, another review by Meltzer and Schuldiner discusses the involvement of CAMs in neuronal remodeling. As a sharp contrast to these broad reviews, and as a reflection of recent surprising developments, László and Lele tell everything you wanted to know about N-cadherin in neural development and disease. An important general issue is the fine balancing of activities controlled by adhesion molecules. This includes not only the positive regulation of cell-cell contacts, but can also involve negative activities. Here, Baeriswyl et al. characterize such balance between positive and negative action in the context of Purkinje cell migration. Two reports focus on the teneurin family of cell adhesion molecules. A review by Dodsworth and Lovejoy focuses on the teneurin C-terminal associated peptides (TCAP), which are encoded by the last exon of teneurins. Interestingly, despite a general transsynaptic interaction of full-length teneurins with latrophilins, evidence suggests that released TCAP molecules have an additional binding capacity to these partners and might elicit distinct cellular process. The presence of teneurins at synapses and their ability of heterocomplexes in cis is described in an article by Cheung et al. The results suggest that the diversity of molecular complexes at synaptic localizations is bigger than previously thought, which thus would increase the combinatoric power to control synaptic specificity. Finally, the process of how synapse formation is controlled through structural domains of different proteins across species is presented in an article by  González-Calvo et al. therefore enabling us to recognize the evolutionary conservation of these fundamental processes.

It is evident that many open questions about the structure and roles of cell adhesion molecules still exist. However, the recent progresses made are encouraging and point toward a better understanding not only in biochemical and cell biological terms, but importantly also in the context of disorders, where there is a clear need for the development of novel therapeutic strategies.

## Author contributions

RH and ZL wrote the summary. Both authors contributed to the article and approved the submitted version.
